# Mitigating overuse of antinuclear antibody (ANA) testing through educational intervention: a study in internal medicine and neurology departments

**DOI:** 10.1007/s10067-024-07180-3

**Published:** 2024-10-16

**Authors:** Yael Pri-Paz Basson, Eran Neumark, Shaye Kivity, Oshrat E. Tayer-Shifman

**Affiliations:** 1 Rheumatology Unit, Meir Medical Center, 59 Tchernichovsky St., 4428164 Kfar Saba, Israel; 2 Faculty of Medical and Health Sciences, Tel Aviv University, Tel Aviv, Israel; 3 Biochemical Laboratory, Meir Medical Center, Kfar Saba, Israel

**Keywords:** Antinuclear antibody (ANA) testing, Choosing wisely, Educational intervention, Healthcare cost reduction, Quality improvement

## Abstract

**Introduction/objectives:**

Overuse of antinuclear antibody (ANA) tests leads to increased costs, false positives, and unnecessary treatments. This study evaluated ANA overuse in internal medicine and neurology departments and assessed the impact of an educational intervention.

**Method:**

This quality improvement educational intervention study examined ANA test overuse in five internal medicine departments and one neurology department at a university-affiliated medical center. The educational intervention included a session focusing on ANA testing appropriateness. Outcome measures comprised the ANA/new patient ratio (APR) and the percentage of positive ANA test results. Outcomes were compared between the pre- and post-intervention periods (both 6 months).

**Results:**

The intervention took place in December 2021. The APR decreased from 43% in the pre-educational intervention period to 27% in the post-intervention period in the neurology department (odds ratio [OR] 0.49, confidence interval [95% CI] 0.37–0.63, *P* < 0.0001) and from 2.6% to 2.2% in the internal medicine departments (OR 0.89, 95% CI 0.73–1.10, *P* = 0.28). The percentage of positive ANA tests increased from 43% pre-intervention to 53% in the post-intervention period (OR 1.49, 95% CI 0.90–2.46, *P* = 0.12) in the neurology department and from 48% to 59% (OR 1.56, 95% CI 0.99–2.44, *P* = 0.0543) in the internal medicine departments.

**Conclusion:**

A simple educational intervention reduced unnecessary ANA testing in the neurology department but not in internal medicine departments, improving patient selection and potential cost savings. The results underscore the importance of targeted education to promote evidence-based behavior among healthcare professionals. Further research with longer follow-up is needed to assess the sustainability of these improvements.

**Supplementary Information:**

The online version contains supplementary material available at 10.1007/s10067-024-07180-3.

**Table:** 

**Key Points** *• This study presents a quality improvement intervention to prevent the overuse of ANA testing in non-rheumatologist physicians.* *• A straightforward educational intervention effectively reduced unnecessary ANA testing and improved patient selection, following the Choosing Wisely campaign.* *• This educational intervention can be adapted and applied in various clinical settings to address the overuse of diagnostic tests, benefiting a wide range of healthcare professionals.*	

## Introduction

Antinuclear antibodies (ANAs) directed against a variety of nuclear antigens have been detected in the serum of patients with systemic autoimmune rheumatic diseases and are an important diagnostic marker for patients with systemic lupus erythematosus (SLE) and other ANA-related autoimmune diseases, such as systemic sclerosis, inflammatory myopathies, mixed connective tissue disease, and Sjögren syndrome [1, 2]. However, these antibodies may be detected in the serum of patients with nonrheumatic diseases, as well as in patients with no definable clinical syndrome [3]. Among healthy people, 25–30% are reported to have positive ANA tests at a titer of 1:40, 10–15% at 1:80, and 5% at 1:160 or greater. This frequency increases with age, particularly in women [3–5]. The interpretation of the ANA test results depends on the pre-test probability of the disease [2].

Inappropriate testing leads to increased healthcare costs and laboratory workload, as well as increased numbers of false-positive results, causing incorrect diagnoses, unnecessary patient anxiety, redundant rheumatology referrals, and even inappropriate treatments [6–8]. In recent years, eliminating unnecessary medical care has received increasing attention from healthcare systems. Choosing Wisely is a campaign that started in the USA and expanded internationally to decrease unnecessary care by focusing on the value of care and potential risks to patients [9]. As part of the Choosing Wisely campaign, the American College of Rheumatology published a list of five things that physicians and patients should question. The first item on this list is “Do not test for antinuclear antibody (ANA) sub-serologies without a positive ANA and clinical suspicion of immune-mediated disease” [5]. This was adopted in the Choosing Wisely campaign in other countries like Canada, which recommended “Don't order anti-nuclear antibodies (ANA) as a screening test in patients without specific signs or symptoms of systemic lupus erythematosus (SLE) or another connective tissue disease (CTD)” [10]. A study in Alberta, Canada, found that although more than 80% of rheumatologists adhere to these recommendations, 1 in 65 citizens have their ANA tested yearly, ordered mainly by non-specialists [11].

We conducted a quality improvement educational intervention based on the Model for Improvement developed by Associates in Process Improvement [12]. It was intended to reduce physicians’ overuse of ANA testing in internal medicine and neurology departments in an academic medical center.

## Methods

### Study design and participants

A quality improvement education intervention was conducted at Meir Medical Center, affiliated with Tel Aviv University. It was intended to educate staff about the appropriateness of ANA testing and reduce the overuse of ANA tests in five Internal Medicine and one Neurology departments. Meir Medical Center in Kfar Saba, Israel, has approximately 800 beds. Some of the departments focus on specific patient illnesses. For example, department C has a hepatology orientation; department E, rheumatology; and department A, hematology. Additionally, the neurology department, which routinely orders ANA tests for hospitalized patients presenting with conditions such as suspected vasculitis, stroke in young individuals, and suspected neuropathy, among others, was included. The study spanned a pre-intervention and a post-intervention period, each lasting 6 months.

### Educational intervention

The quality improvement educational intervention took place in December 2021. It was structured as a 30-min group session with various personnel from the departments participating, including 2-3 senior hospitalists specializing in internal medicine, 7-8 residents, and the department head. The same educational intervention was presented individually in all six participating departments. Led by a certified rheumatologist (OTS), with additional support from other certified rheumatologists (YPB, SK), these sessions provided physicians with comprehensive insights into ANA testing. The content covered explanations of laboratory procedures, guidance on appropriate circumstances for requesting ANA testing in routine clinical settings, and the application of Choosing Wisely guidelines for the test (for presentation, see supplementary). During the session, it was highlighted that the ANA test has low specificity and should not be used as a screening test in patients without specific signs or symptoms of SLE or other connective tissue diseases. Additionally, it was noted that when the ANA test is used in cases with a low predictive probability of ANA-associated diseases based on clinical manifestations, there are more false-positive results, leading to overdiagnosis and overtreatment. At the end of the structured presentation, department-specific information was communicated, including insights into their ANA test utilization, presentation of outcome metrics, and relevant comparative data with other participating departments. Past clinical cases involving ANA testing were discussed with the staff of each department. Each department was requested to continue discussing this issue during the next morning’s meetings. Additionally, they were asked to introduce the intervention session to staff members who were absent due to night duty or vacation. The issue continued to be discussed during rheumatology consultations in the departments.

### ANA testing and positivity

All ANA testing was done in the Biochemical Laboratory at Meir Medical Center. ANA testing was done using the indirect immunofluorescence method on HeP-2 cells, according to international guidelines [3]. A positive result was defined as a titer of 1:160 or higher.

### Outcome measures

Outcome measures were the ANA/new patient ratio (APR) to assess the number of ANA tests ordered, and the percentage of positive ANA tests was used to evaluate the appropriateness of patient selection; both were measured 6 months pre-intervention and 6 months post-intervention. The percentage of repeat ANA tests was measured in the internal medicine and the neurology departments 1-year pre-intervention and 1-year post-intervention.

### Data sources

Laboratory databases from Meir Medical Center were used to retrieve ANA data. These included the number of ANA tests performed, test results, and test dates. Information on the number of new patients hospitalized in the specific department was obtained from administrative hospital databases. Patient data were analyzed anonymously.

All ANA test data were retrieved from the relevant sources at two time points: 6 months pre-intervention and 6 months after the educational intervention. The data retrieved from the pre-intervention period were used to prepare the feedback provided to the departments during the intervention. The data were obtained retrospectively.

### Ethical considerations

The protocol was reviewed by the Institutional Review Board of Meir Medical Center, and as it was a qualitative study involving medical staff, it did not require approval.

### Statistical analysis and reporting of results

Outcome measures were compared between the pre- and post-intervention periods. All are reported as odds ratios (ORs) with the corresponding 95% confidence intervals (95% CI) and *P* values. Data were analyzed with SPSS version 27 (IBM Corporation, Armonk, NY).

## Results

**Fig. 1:**
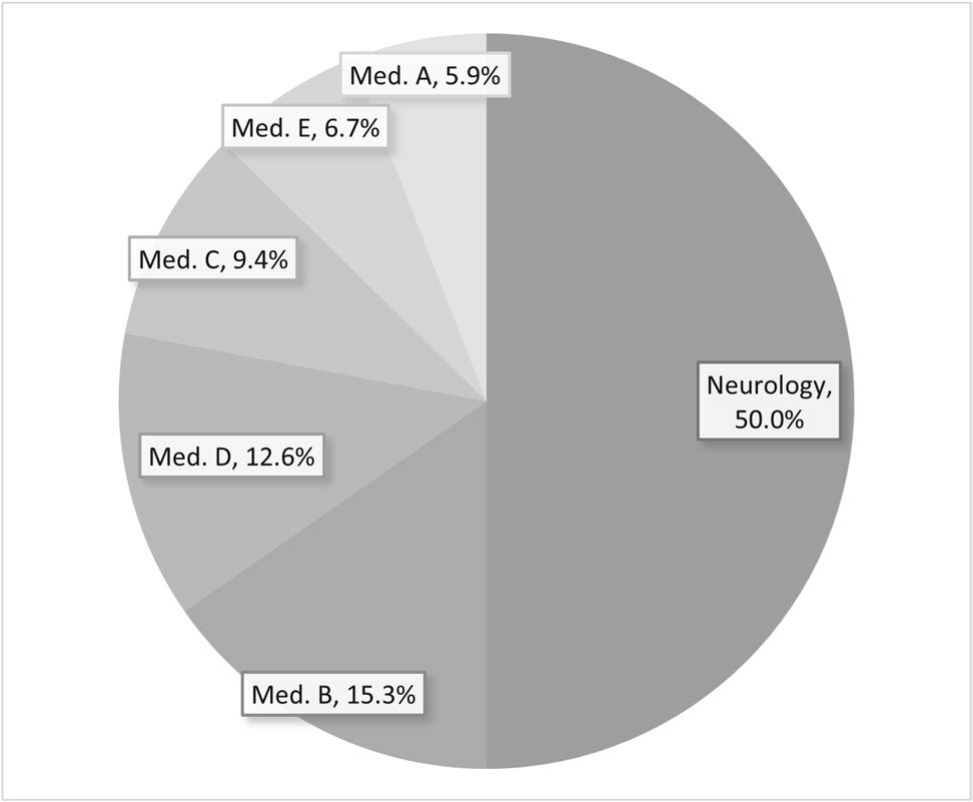
Percentage of ANA test ordered by each department in the pre- educational intervention period

In the pre-educational intervention period, 372 ANA tests were ordered by the internal medicine and neurology departments. The neurology department requested half and the rest from the five internal medicine departments (Fig. 1).

### ANA/new patient ratio (APR)

**Fig. 2:**
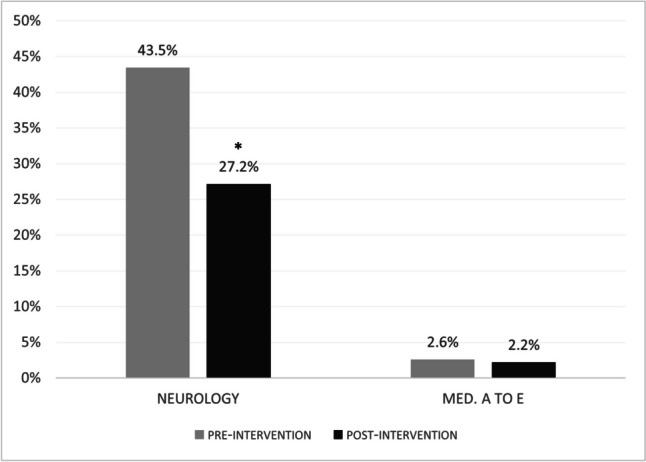
ANA to new patient ratio (APR) in the pre- and post-educational intervention periods in the neurology and internal medicine departments

During the pre-educational intervention period, the APR in the neurology department was 43%. The APR decreased in the post-educational intervention period to 27%, with an OR of 0.49 (95% CI 0.37–0.63, *P* < 0.0001). In the internal medicine departments, it decreased from 2.6% to 2.2%, with an OR of 0.89 (95% CI 0.73–1.10, *P* = 0.28) (Fig. 2).

### Percentage of positive ANA tests

**Fig. 3:**
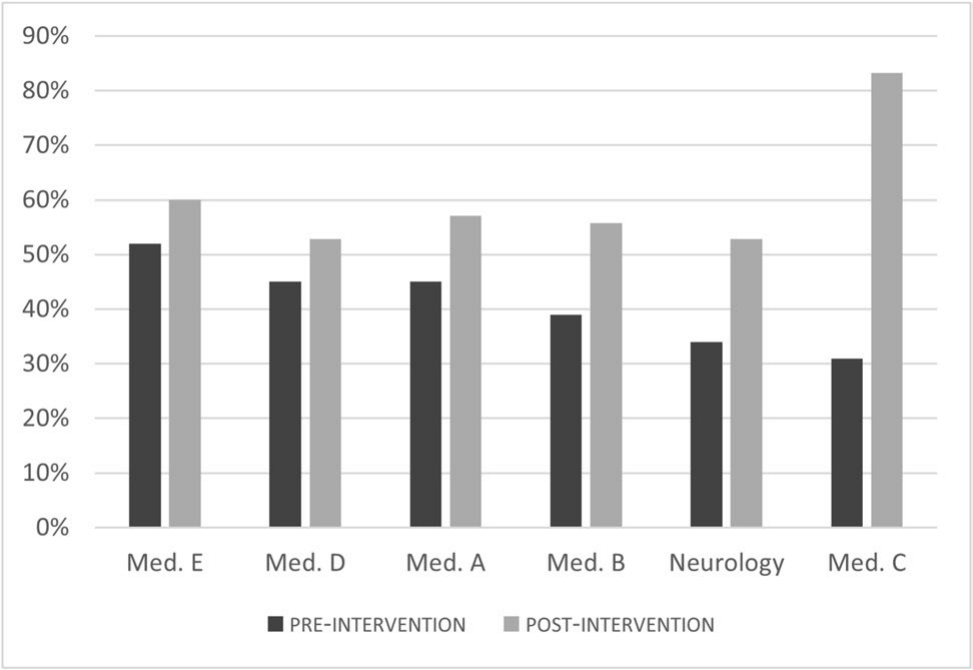
Percentage of positive ANA tests according to department in the pre- and post-educational intervention periods

The percentage of positive ANA tests in the neurology department increased from 43% in the pre-educational intervention period to 53% in the post-intervention period, with OR 1.49 (95% CI 0.90–2.46, *P* = 0.12). In the internal medicine departments, it increased from 48% to 59%, OR 1.56 (95% CI 0.99–2.44, *P* = 0.0543), overall. An increase in the percentage of positive ANA tests was observed in the individual internal medicine departments as well (Fig. 3).

### Percentage of repeated ANA tests

The percentage of repeat ANA tests in the internal medicine departments and the neurology department was 3.4% in the year before the educational intervention and 2.5% in the year after the educational intervention.

## Discussion

This study described a quality improvement educational intervention intended to improve the appropriateness of ANA testing in the neurology and internal medicine departments in our hospital.

We demonstrated that a straightforward educational intervention was associated with a decrease in the number of ANA tests ordered, as reflected by the APR, and an improvement in patient selection, as reflected by an increase in the proportion of positive ANA test results. Specifically, there was a 50% reduction in ANA tests ordered by the neurology department, which had previously accounted for a substantial portion of all ANA tests. While the decrease in ANA tests ordered by the internal medicine departments was more modest and did not reach statistical significance, this trend was observed consistently across all five departments. Moreover, the departments exhibited enhanced patient selection, indicated by an increase in the percentage of positive test results from 48% to 59% (*P* = 0.054).

In a study that evaluated utilization patterns, appropriateness, and associated costs of tests in patients referred to rheumatologists, ANA testing was the most frequently ordered investigation and was most likely to be ordered inappropriately, with a low positive result ratio [8]. Barry et al. found that by changing the options in the electronic health record, the ordering patterns shifted toward Choosing Wisely recommendations [12].

Lesuis et al. [13] showed that a relatively simple intervention improved rheumatologists’ behavior in requesting ANA tests. The intervention was a 1-h group session for rheumatologists in three centers, combining an educational meeting with feedback. Six months after the intervention, a booster session was held. They found a significant decrease in the number of ANA tests requested in the post-intervention period. Comparable to our findings, there was a decrease in APR, but unlike our study, Lesuis et al. did not find an increase in the percentage of positive ANA tests. They had a second session and a longer follow-up period than we did and found a decrease in repeat ANA requests after the additional session.

We observed a slight decrease in the number of repeat ANA tests in the year following the educational intervention. However, it is essential to note that we cannot definitively attribute this decrease to the educational intervention alone, as there were also changes in how the departments ordered tests during this year.

Nevertheless, the ANA test is predominantly ordered by primary care physicians [7] along with a diverse range of clinicians, including internists, dermatologists, nephrologists, neurologists, and gynecologists [2]. Because most ANA tests in our hospital were ordered by the neurology and internal medicine departments, an intervention to improve overall ANA test–requesting behavior was necessary and showed encouraging results.

This study had several limitations. It was not a controlled intervention but rather an analysis of the differences in ratios of tests ordered and positive results before and after an educational intervention. Additionally, the departments are heterogeneous, making direct comparisons challenging. For example, internal medicine department C focuses on hepatology, leading to more frequent ANA test requests when autoimmune hepatitis is suspected. In contrast, internal medicine department E has a rheumatology orientation and tends to repeat ANA testing in patients with known systemic autoimmune rheumatic diseases. Unfortunately, we could not retrieve information about which doctor ordered which test. We did not collect individualized patient data on the reason for hospitalization, patients’ diagnoses at discharge, and other clinical information necessary for estimating pre-test probability. Conducting detailed chart reviews would have been resource-intensive. Consequently, we could not directly demonstrate that our educational intervention improved patient selection and did not result in misdiagnoses due to the reduction in ANA testing. However, previous research by Ferrari indicated that the risk of missing a diagnosis of SLE is very low when ANA tests are conducted according to the Canadian Rheumatology Association’s recommendations, which advise against ordering antibody serology unless specific signs and symptoms of SLE are present [10]. Therefore, the likelihood of underdiagnosing ANA-associated diseases was minimal. Lastly, our follow-up period was limited to 6 months, and longer-term monitoring is necessary to assess the sustainability of our implemented changes.

Despite its limitations, the favorable outcomes found in this study demonstrate that a simple educational intervention can enhance decision-making and reduce excessive ANA testing and associated laboratory work, which in turn may lead to improved diagnostic and treatment processes.

## Supplementary Information

Below is the link to the electronic supplementary material.

Supplementary file1 (PDF 497 KB)

## Data Availability

The data that support the findings of this study are available on request from the corresponding author.
