# Supplementary material for: Mitigating overuse of antinuclear antibody (ANA) testing through educational intervention: a study in internal medicine and neurology departments
Source: Clin Rheumatol. 2024 Oct 16;43(12):3935–9. doi: 10.1007/s10067-024-07180-3 (PMC11582184; doi:10.1007/s10067-024-07180-3)
Supplement: Supplementary file 1 — Supplementary file1 (PDF 497 KB) [file 10067_2024_7180_MOESM1_ESM.pdf]

Choosing Wisely: ANA

Oshrat Tayer-Shifman
Rheumatology Unit
Meir Medical Center
Kfar Saba, Israel

What are ANAs?

Anti-nuclear antibodies including among others:

- Anti-dsDNA
- Anti-RNP
- Anti-Ro (SS-A)
- Anti-La (SS-B)
- Anti-Sm
- Anti-SCL-70
- Anti-centromere
- Anti-Jo-1, etc.

Sensitive for some rheumatic diseases
But not specific...

Diseases associated with a positive ANA
Infectious diseases:<br>Viral (EBV, HIV, HCV, parvovirus 19)<br>Bacterial (SBE, syphilis)
Malignancies:<br>lymphoproliferative disease<br>paraneoplastic syndromes
Miscellaneous:<br>inflammatory bowel disease<br>interstitial pulmonary fibrosis

EBV: Epstein-Barr virus; HCV: hepatitis C virus; SBE: subacute bacterial endocarditis

UpToDate®

Frequency of ANAs in autoimmune diseases	Sensitivity (%)
Systemic lupus erythematosus	95-100
Systemic sclerosis	60-80
Mixed connective tissue disease	100
Inflammatory myopathies	60
Rheumatoid arthritis	50
Sjogren syndrome	30-50
Discoid lupus	15
Pauciarticular JIA	70

Hochberg MC, Silman AJ, Smolen JS, Weinblatt ME, Weisman MH, Gravallese EM. Rheumatology: Elsevier Health Sciences; 2018.

In healthy individuals

ARTHRITIS & RHEUMATISM
Vol. 40, No. 9, September 1997, pp 1601–1611
© 1997, American College of Rheumatology

RANGE OF ANTINUCLEAR ANTIBODIES IN "HEALTHY" INDIVIDUALS

E. M. TAN, T. E. W. FELTKAMP, J. S. SMOLEN, B. BUTCHER, R. DAWKINS, M. J. FRITZLER, T. GORDON, J. A. HARDIN, J. R. KALDEN, R. G. LAHITA, R. N. MAINI, J. S. McDOUGAL, N. F. ROTHFIELD, R. J. SMEENK, Y. TAKASAKI, A. WIIK, M. R. WILSON, and J. A. KOZIOL

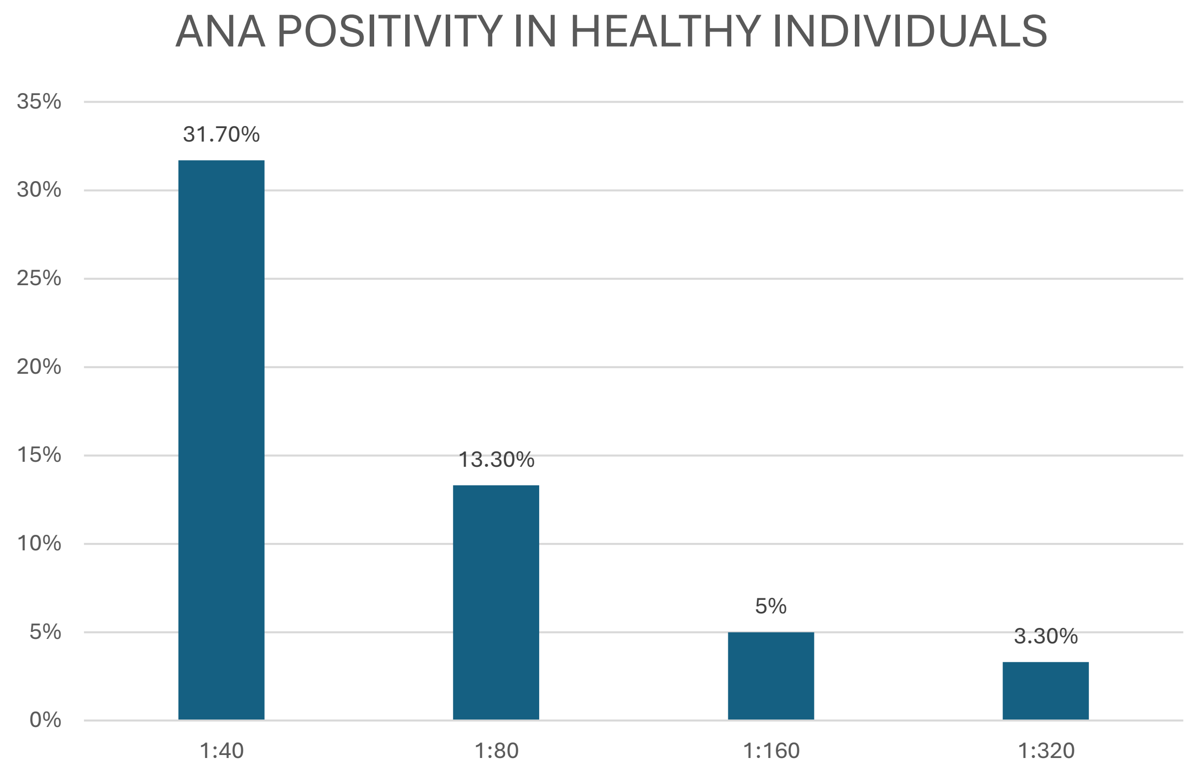

Increased frequency:

- Relatives of patients with autoimmune diseases
- Individuals receiving certain medications
- Elderly

Tan EM, Feltkamp TE, Smolen JS, Butcher B, Dawkins R, Fritzler MJ, et al. Range of antinuclear antibodies in "healthy" individuals. Arthritis Rheum. 1997;40(9):1601-11.

How is ANA testing performed?

1. Indirect immunofluorescence (IFA):

   Sensitive, time consuming

2. Solid phase assays:

   Screening test for specific Antigens

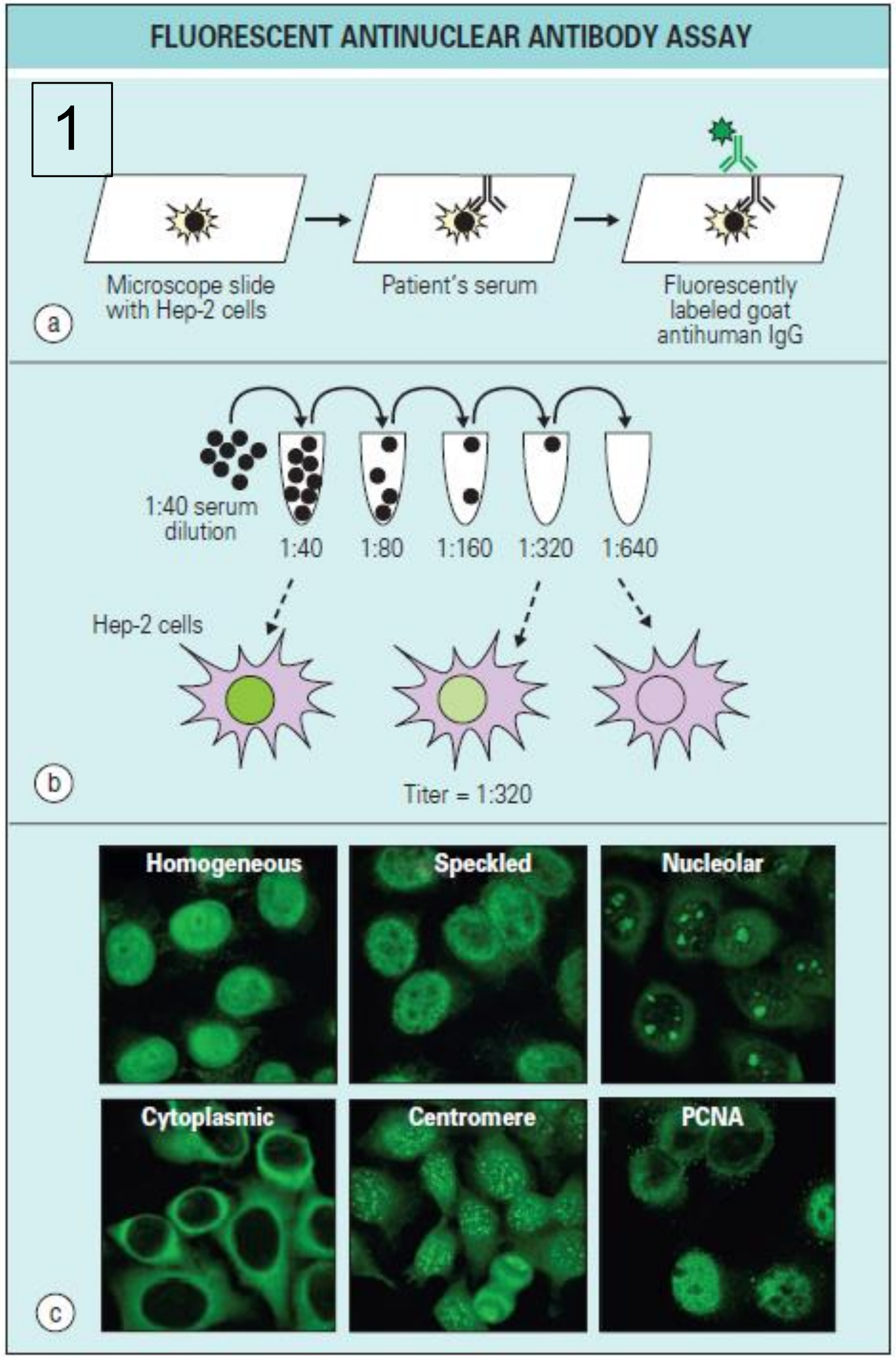

Hochberg MC, Silman AJ, Smolen JS, Weinblatt ME, Weisman MH, Gravallese EM. Rheumatology: Elsevier Health Sciences; 2018.

Which patients should we send for ANA testing?

Arthritis & Rheumatism (Arthritis Care & Research)
Vol. 47, No. 4, August 15, 2002, pp 434–444
DOI 10.1002/art.10561
© 2002, American College of Rheumatology

SPECIAL ARTICLE

Evidence-Based Guidelines for the Use of Immunologic Tests: Antinuclear Antibody Testing

DANIEL H. SOLOMON,[1] ARTHUR J. KAVANAUGH,[2] PETER H. SCHUR,[1] AND THE AMERICAN COLLEGE OF RHEUMATOLOGY AD HOC COMMITTEE ON IMMUNOLOGIC TESTING GUIDELINES

Table 10. Conditions associated with a positive antinuclear antibody (ANA)

ANA very useful for diagnosis
Systemic lupus erythematosus
Systemic sclerosis
ANA somewhat useful for diagnosis
Sjögren's syndrome
Polymyositis-dermatomyositis
ANA very useful for monitoring or prognosis
Juvenile chronic arthritis
Raynaud's phenomenon
ANA is a critical part of the diagnostic criteria
Drug-associated lupus
Mixed connective tissue disease
Autoimmune hepatitis
ANA not useful or has no proven value for diagnosis, monitoring or prognosis
Rheumatoid arthritis
Multiple sclerosis
Thyroid disease
Infectious disease
Idiopathic thrombocytopenic purpura
Fibromyalgia

Solomon DH, Kavanaugh AJ, Schur PH. Evidence-based guidelines for the use of immunologic tests: antinuclear antibody testing. Arthritis Rheum. 2002;47(4):434-44.

CHOOSING WISELY CANADA

- “Do not order antinuclear antibodies (ANA) as a screening test in patients without specific signs or symptoms of systemic lupus erythematosus (SLE) or another connective tissue disease (CTD).”
  - ANA testing should not be used to screen subjects without specific symptoms (e.g., photosensitivity, malar rash, symmetrical polyarthritis, etc.), or without a clinical evaluation that may lead to a presumptive diagnosis of SLE or other connective tissue disease…
  - In a patient with low pretest probability for ANA-associated rheumatic disease, positive ANA results can be misleading and may precipitate further unnecessary testing, erroneous diagnosis, or even inappropriate therapy.”
  - Repeat ANA adds little if any clinical value to patient management such as monitoring disease activity, confirming remission or predicting disease flares (adults)

Ferrari R. Evaluation of the Canadian Rheumatology Association Choosing Wisely recommendation concerning anti-nuclear antibody (ANA) testing. Clin Rheumatol. 2015;34(9):1551-6.

Combination of ≥2 signs/symptoms suggesting SLE: Request ANA

- Typical rash
- Oral ulcers
- ≥ 2 joints arthritis or morning stiffness
- Serositis/pleuritis/pericarditis
- Kidney disease
- Typical neurologic involvement
- Cytopenia
- Anti-phospholipid Antibodies
- Alopecia
- Low complement

Ferrari R. Evaluation of the Canadian Rheumatology Association Choosing Wisely recommendation concerning anti-nuclear antibody (ANA) testing. *Clin Rheumatol* 2015. doi: 10.1007/s10067-015-2985-z

Why not send everyone for ANA testing ?

1. Uncertainty and anxiety (doctors and patients)
2. Additional unnecessary tests
3. Over diagnosis, over treatment
4. Lab overload
5. Cost

Cost

Table 2
HCE ANA testing and AARD case finding (per million population)*,**, ***

Component/assumption	Numeric value	Cost (CDN $)	Percent of total HCE/TOTAL DIE	Action/comments
Average per person HCE Canada	$6000	6 Billion	100	
Annual ANA test rate 1/250	4000 tests @$15,00.test	60,000		ANA test on HEp-2 substrates at serum dilution of 1/80
Retest and titer (25% of tests)	1000 tests @ $15.00/test	15,000		Samples are retested to determine titer or retested due to technical considerations
Total ANA test cost		75,000	0.00125	
200 ANA positive patients referred or further evaluated	200**			Referral rate of ANA positive patients reported at 20% = 200 patients referred
Consultants fee	$125	25,000		
60% of 200 (120) patients evaluated further by lab tests including ANA Subserology	$350.00	42,000		For cost breakdown for further evaluation refer to Table 3.
50% (60 patients) seen in follow-up	$100.00	6000		
TOTAL HCE CASE FINDING AARD		73,000		
TOTAL INCLUDING ANA and ANA SUBSEROLOGY TESTING	$75,000	148,000	0.00247	

Abbreviations: AARD, ANA, anti-nuclear antibody; ANA related rheumatic disease; DIE, Diagnostic Investigation Expenditures; HCE, Health Care Expenditures (total).
* Based on Alberta Canada.
** Based on published referral pattern of ANA positive patients [28].
*** Elements and costs included in AARD investigation are detailed in Table 3.

Fritzler MJ. Choosing wisely: Review and commentary on anti-nuclear antibody (ANA) testing. Autoimmun Rev. 2016;15(3):272-80.

Conclusions

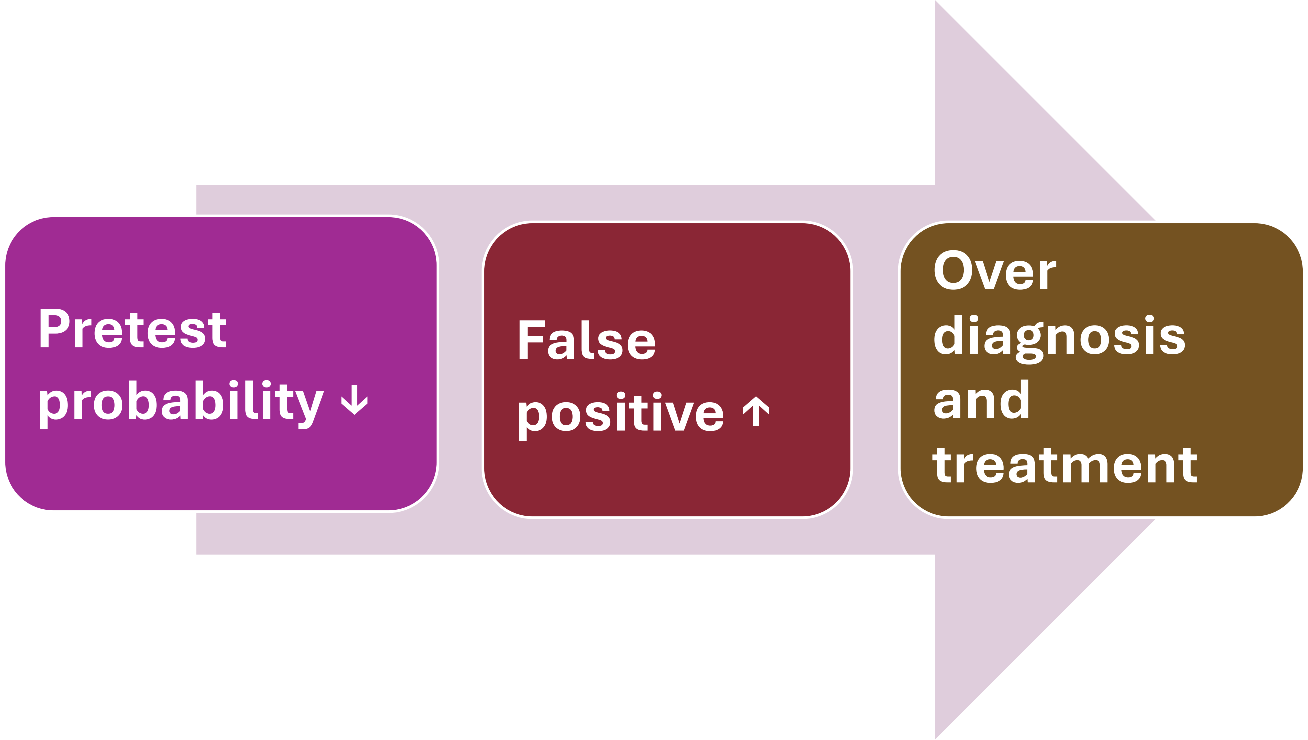

Send ANA only when thinking of:

1. SLE
2. Systemic sclerosis (scleroderma)
3. Sjogren syndrome
4. Polymyositis/ dermatomyositis
5. Raynaud's syndrome
6. Mixed connective tissue disease
7. Autoimmune hepatitis

Cases
